# Tranexamic Acid for Postpartum Hemorrhage Treatment in Low-Resource Settings: A Rapid Scoping Review

**DOI:** 10.3390/ijerph19127385

**Published:** 2022-06-16

**Authors:** Nguyen Toan Tran, Sarah Bar-Zeev, Catrin Schulte-Hillen, Willibald Zeck

**Affiliations:** 1 Australian Centre for Public and Population Health Research, Faculty of Health, University of Technology Sydney, Sydney, NSW 2007, Australia; 2 Faculty of Medicine, University of Geneva, 1211 Geneva, Switzerland; 3 United Nations Population Fund, Technical Division, New York, NY 10158, USA; bar-zeev@unfpa.org (S.B.-Z.); zeck@unfpa.org (W.Z.); 4 United Nations Population Fund, Humanitarian Office, 1202 Geneva, Switzerland; schulte-hillen@unfpa.org

**Keywords:** tranexamic acid, postpartum hemorrhage, treatment, health system, low-resource settings, scoping review

## Abstract

Tranexamic acid (TXA) effectively reduces bleeding in women with postpartum hemorrhage (PPH) in hospital settings. To guide policies and practices, this rapid scoping review undertaken by two reviewers aimed to examine how TXA is utilized in lower-level maternity care settings in low-resource settings. Articles were searched in EMBASE, MEDLINE, Emcare, the Maternity and Infant Care Database, the Joanna Briggs Institute Evidence-Based Practice Database, and the Cochrane Library from January 2011 to September 2021. We included non-randomized and randomized research looking at the feasibility, acceptability, and health system implications in low- and lower-middle-income countries. Relevant information was retrieved using pre-tested forms. Findings were descriptively synthesized. Out of 129 identified citations, 23 records were eligible for inclusion, including 20 TXA effectiveness studies, two economic evaluations, and one mortality modeling. Except for the latter, all the studies were conducted in lower-middle-income countries and most occurred in tertiary referral hospitals. When compared to placebo or other medications, TXA was found effective in both treating and preventing PPH during vaginal and cesarean delivery. If made available in home and clinic settings, it can reduce PPH-related mortality. TXA could be cost-effective when used with non-surgical interventions to treat refractory PPH. Capacity building of service providers appears to need time-intensive training and supportive monitoring. No studies were exploring TXA acceptability from the standpoint of providers, as well as the implications for health governance and information systems. There is a scarcity of information on how to prepare the health system and services to incorporate TXA in lower-level maternity care facilities in low-resource settings. Implementation research is critically needed to assist practitioners and decision-makers in establishing a TXA-inclusive PPH treatment package to reduce PPH-related death and disability.

## 1. Introduction

Primary postpartum hemorrhage (PPH) is usually defined as bleeding within the first 24 h equaling 500 mL or more after vaginal birth and 1000 mL or more after cesarean birth [1]. It is the major cause of maternal mortality globally and accounts for around 100,000 deaths each year [2]. Almost all the mortality burden falls on low-income and middle-income countries [3].

The causes of primary PPH include uterine atony, retained placenta, trauma, uterine rupture, clotting disorders, and uterine inversion [4]. Most deaths occur shortly after birth, and, therefore, prompt management of PPH is critical [5]. The World Health Organization (WHO) recommends a multidisciplinary approach with a first line of actions that includes fluid replacement and a repeated dose of uterotonic (an initial uterotonic dose should have been administered as part of PPH prevention measures) [1,6]. 

Tranexamic acid (TXA), an antifibrinolytic agent or clot stabilizer, reduces the risk of bleeding in surgery and trauma [7]. From a health system perspective, it is not limited by specific storage requirements, such as cold chain, and is often available in hospital settings because of its use in surgery and trauma. It is relatively affordable at $1.30 per 1 g ampoule (www.unfpaprocurement.org/products, accessed on 8 November 2021). Applied to obstetric care, TXA effectively reduces bleeding in women with PPH and, from a clinical perspective, has the advantage to do so, irrespective of the underlying diagnosis [8].

The effectiveness of TXA for PPH treatment has been established in recent studies and systematic reviews. In their 2018 Cochrane Review, Shakur et al. concluded that intravenous TXA given immediately after bleeding onset reduces primary PPH mortality by 19% in vaginal and cesarean births without increased thromboembolic risk [9]. In terms of secondary outcomes, TXA did not reduce the risk of hysterectomy to control bleeding, serious maternal morbidity, or blood transfusion. Over 99% of the Cochrane Review data originated from the landmark WOMAN trial, which took place in the hospital settings in 21 low-, middle-, and high-income countries [8]. Low-income countries comprised Burkina Faso, the Democratic Republic of Congo, Ethiopia, Sudan, and Uganda. In their 2021 systematic review of the cost-effectiveness of TXA for PPH treatment, Aziz identified four studies from three countries [10]. They concluded that TXA is cost-effective in Nigeria and Pakistan but not in the United States unless the probability of death due to PPH is high enough. In other words, TXA is likely to be cost-effective in situations where PPH and PPH-related morbidity are prevalent and TXA is available at a low price.

Regarding the effectiveness of TXA for preventing PPH, Novikova found in their 2015 Cochrane Review that TXA, in addition to uterotonics, decreases postpartum bleeding and prevents PPH and blood transfusions among women at low PPH risk undergoing spontaneous and elective cesarean birth [11]. However, the review was based on mixed-quality evidence. The review also found insufficient evidence to draw conclusions about thromboembolic events, mortality, TXA use in high-risk women, and severe side-effects. However, an increase in minor side-effects related to TXA use was identified.

Following the WOMAN trial, the WHO issued the following recommendation on the use of TXA for PPH treatment: women diagnosed clinically with PPH after vaginal or cesarean birth should receive within 3 h of delivery a fixed dose of 1 g (100 mg/mL) intravenously at 1 mL per minute (over 10 min); this is followed by a second dose of 1 g if bleeding continues after 30 min or if bleeding resumes within 24 h after the first dose [12]. Subsequently, the 2019 edition of the WHO Model List of Essential Medicines included TXA for PPH treatment into the core list of reproductive health medicines [13]. Based on extant literature, the WHO does not recommend TXA for PPH prevention and has prioritized studying this topic [11].

In addition to the evidence on TXA effectiveness for PPH treatment, policymakers and program managers require other information to support its adoption and implementation, especially in resource-constrained settings, where the highest maternal mortality is concentrated. The feasibility at different levels of maternity care, acceptability by providers and women, and health system requirements, such as policy changes, staff capacity, and procurement and storage issues, are all examples of such information. There are no published reviews on this research subject to our knowledge. Therefore, a compilation of best practices and implementation experiences from resource-constrained contexts might be helpful and constitute the primary objective of our scoping review.

## 2. Materials and Methods

We undertook a rapid scoping review of the literature to summarize recent TXA implementation practices and health-system implications. A rapid review synthesizes knowledge by omitting or reducing elements of the systematic review process in order to collect information in a shorter time [14]. Scoping reviews aim to provide academics, decision-makers, and practitioners with an overview of a topic in order to identify key concepts, evidence types, and knowledge gaps in developing issues [15]. The rapid scoping review was completed in eight weeks by two reviewers. We used the PRISMA statement to report the outcomes (see Appendix A).

### 2.1. Protocol

A protocol was established and can be accessed on the Open Science Framework (https://osf.io/z47ep/).

### 2.2. Literature Search Strategy

We used Ovid to search the following five bibliographic databases: EMBASE, MEDLINE, Emcare, the Maternity and Infant Care Database (MIDIRS), and the Joanna Briggs Institute Evidence-Based Practice (JBI EBP) Database. In addition, the Cochrane Library was searched using *tranexamic acid*, *postpartum hemorrhage*, and *developing countries* as the main search terms. *Developing countries* is a Medical Subject Heading (MeSH) term in MEDLINE and was used as a proxy for low-resource settings. We also broadened the search to include all countries, which the World Bank classifies as low-income or lower-middle-income (Appendix A). The search was limited to publications published between 1 January 2011 and 15 September 2021 in English, French, and Spanish. The final MEDLINE search procedure is available in Appendix A. EndNote was used to remove duplicates.

### 2.3. Eligibility Criteria

The first round of screening consisted of a review of the titles and abstracts. The criteria in Table 1 were used to find potential papers and evaluate them for inclusion, guiding the final selection of full-text publications. In addition to the research design, the population, concept, and context (PCC) framework was used to create the eligibility criteria. The JBI recommends the PCC framework for scoping reviews as a better option than the population-intervention-comparator-outcome mnemonic used in systematic reviews [16]. 

### 2.4. Study Selection and Data Abstraction

For additional screening, we imported non-duplicated papers into JBI SUMARI. We reviewed the full texts of eligible publications after the titles and abstracts were evaluated for eligibility. The data from the studies that were included were retrieved using tables pilot-tested on two random publications. Study characteristics were included in one table (e.g., first author, publication year, location, aim, method, study population, intervention type and outcome measures, and relevant findings). We created another table to summarize concepts related to our study topic, such as country income categorization, levels of healthcare (basic (BEmOC) vs. comprehensive (CEmOC) emergency obstetric care), and desired outcomes (feasibility, acceptability, and effectiveness). Effectiveness was also included because it was a recurrent characteristic among the identified papers and allowed comparison with the other outcomes. Health system considerations were also included, using the WHO framework of the six-health system building blocks, i.e., (i) government and policy alignment, (ii) health staff awareness, motivation, and training, (iii) service delivery, (iv) procurement channels and commodity security, (v) financing, and (vi) health information system. Two reviewers performed separately the article screening, with a third reviewer resolving any differences.

### 2.5. Methodological Quality Appraisal

There was no assessment of the methodological quality of the included papers, as per standards for conducting scoping reviews [17].

### 2.6. Synthesis

Tables on study characteristics and key programmatic aspects are used to present the review findings in a descriptive form.

## 3. Results

### 3.1. Quantity of Research Available

**Figure 1:**
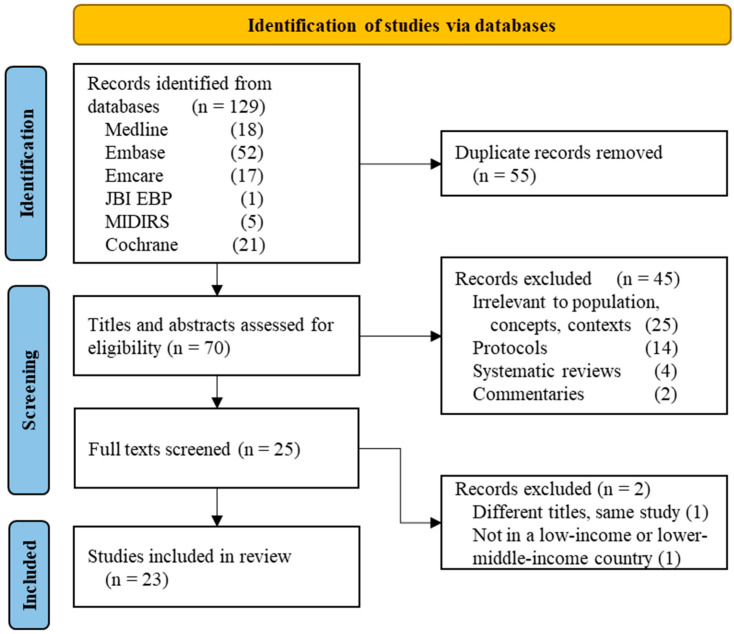
Study flow chart.

We identified 129 citations in the electronic database search, 55 of which were duplicates (Figure 1). Based on titles and abstracts, 45 were eliminated from the remaining 70 because they were commentaries (2), systematic reviews (4), protocols (14), or unrelated to our topic (25). From the 25 records suitable for full-text screening, two were excluded: one was a duplicate of an already-included study but with a different title, and the other did not occur in an eligible country (Thailand). Eight records turned out to be conference abstracts that were not followed by a full-length paper. Based on the relevance of all eight records and the scoping nature of our review, they were nonetheless included in the final set of 23 references.

Table 2 quantitatively summarizes the publication type, study country by income group, study type, and outcome of interest. Two-thirds of the references were full-text articles and one-third conference abstracts. The majority originated from lower-middle-income countries, with India, Iran, and Tunisia among the top three. There were 20 TXA effectiveness studies, two economic evaluations, and one maternal mortality modeling. Acceptability was addressed in one article, operational feasibility in three, and financial feasibility in three.

### 3.2. Study Design

The relevant characteristics of the included studies are synthesized in Table 3.

Out of the 20 studies examining TXA effectiveness, 14 (70%) were randomized controlled trials (RCTs) [18,19,20,21,22,23,24,25,26,27,28,29,30,31] and the rest non-randomized trials [32,33,34,35,36,37].

Overall, the trials tested TXA not only for PPH treatment but also for PPH prevention. While TXA was most of the time compared with routine care and placebo, a few studies used other comparators or combined TXA with other medications, including misoprostol sublingual [20,21], misoprostol per rectum [29], a prostaglandin analog [31,34], and fibrinogen [33].

Among the two economic evaluations, Li examined the cost-effectiveness of early administration of TXA within 3 h after vaginal and cesarean births, using a decision tree and healthcare provider perspectives [38]. Resch developed a cost-effectiveness model to compare three PPH intervention packages: (i) a routine bundle with intravenous fluids, uterotonics, and uterine massage, (ii) a strengthened bundle consisting of the routine bundle plus TXA as well as manual removal of placenta and suturing when indicated, and (iii) and an enhanced bundle reinforcing (ii) with non-surgical interventions for managing refractory PPH [39].

McClure modeled the impact of TXA used for PPH prevention and treatment on PPH-related maternal mortality in sub-Saharan Africa, assuming an overall 30% efficacy of TXA to reduce PPH [40].

**Table 3:** Characteristics of included studies.

Study & Year	Location	Aim	Method/Design	Study Population and Sample Size	Intervention Type & Outcome Measures	Relevant Findings
Abdel-Aleem, 2013 [18]	Women’s Health Hospital, Assiut University, Assiut, Egypt	To assess the possible effect of TXA on blood loss during and after elective c-section	Two-arm non-blinded RCT	740 pregnant women with singleton fetus at ≥37 weeks planned to have elective c-section	TXA 1 g IV, 10 min before c-section Primary outcome: Blood loss measured during and 2 h after operation	Pre-operative use of TXA is associated with reduced blood loss during and after elective c-section. This could benefit anemic women or those who refuse blood transfusion. Mean total blood loss was 241.6 (SE 6.77) mL in the TXA group versus 510 (SE 7.72) mL in the control group. The mean drop in hematocrit and hemoglobin levels were statistically significantly lower in the TXA group than in the control group. There were no statistically or clinically significant differences in other outcomes. The study was not powered to assess the efficacy of TXA in prevention of severe PPH or to assess its safety especially thromboembolic complications.
Agrawal, 2018 [19] (Conference abstract only)	BPKIHS medical university in eastern Nepal	To evaluate the effect of preoperative administration of IV TXA on blood loss during and after elective c-section	RCT, blinding not mentioned	160 pregnant women at ≥37 weeks with elective c-section	TXA 1 g IV vs. normal saline Primary outcome: blood loss during and for 24 h after operation	The mean estimated blood loss was significantly lower in women treated with TXA compared with women in the placebo group (392.13 ± 10.06 mL versus 498.69 ± 15.87 mL)
Ajroudi, 2015 [32] (Conference abstract only)	Mongi Slim Hospital, La Marsa, Tunisia	To assess the efficacy of a new protocol including TXA in the management of PPH	Non-randomized trial	40 women with PPH following vaginal or cesarean delivery	TXA loading dose 1 g/10 min, then infusion of 1 g/h over 3 h, in addition to the classic protocol including oxytocin and prostaglandins Primary outcome: protocol success rate	TXA reduces blood loss and maternal morbidity in PPH: the protocol succeeded in 81.1% of the cases, no adverse effects of TXA, 18% of patients required a blood transfusion.
Bose, 2017 [20]	Hospital-based Malabar Institute of Medical Sciences, Calicut, Kerala, India	To compare misoprostol vs. TXA in reducing blood loss during c-section	RCT, non-blinded	163 pregnant women with emergency/elective c-section Gestational age not mentioned	TXA 500 mg IV vs. misoprostol 600 mcg SL Primary outcome: blood loss reduction, additional uterotonic use	TXA significantly reduced blood loss compared with misoprostol (416 vs. 505 mL, *p* = 0.023) in patients without high-risk factors for PPH, but not in patients with PPH risk factors. Misoprostol caused significantly higher minor side effects while TXA reduced operation time (by 5 min). Medication costs mentioned but cost analysis not done (misoprostol tablet manufactured by Cipla at INR 52 per dose vs. INR 57 per TXA dose, manufactured by Ozone).
Briki, 2018 [33]	Farhat Hached University Hospital, Sousse, Tunisia.	To evaluate the combination of TXA and fibrinogen concentrates in severe PPH	Retrospective observational study	166 women with >24 weeks pregnancy and severe PPH (≥500 mL if vaginal delivery or ≥1000 mL if c-section)	Mean doses: TXA 1.98 g & fibrinogen 2.25 g Primary outcome: blood loss	Significant decrease in the fall of hemoglobin and blood transfusion in intervention group. Hemoglobin levels post-delivery: 6.23 ± 1.56 g/dl for control, 7.31 ± 2.09 for TXA & fibrinogen, *p* = 0.003
Dimassi, 2018 [34] (Conference abstract only)	Moni Slim Hospital, Tunis, Tunisia	To evaluate the results of a therapeutic protocol with TXA and sulprostone for PPH care management	Prospective descriptive study	70 women with PPH after vaginal or cesarean delivery. Gestational age not mentioned	TXA and sulprostone, unknown posology Primary outcome: success rate of protocol	The success rate of medical care management was 87.1%. The mean time for the diagnosis of the bleeding was 32 min.
Diop, 2020 [21]	4 hospitals in Senegal and Vietnam	To evaluate the efficacy, safety, and acceptability of oral TXA when used as an adjunct to sublingual misoprostol to treat PPH following vaginal delivery.	Double-blind RCT	258 women with PPH (defined as ≥700 mL) after vaginal birth. Gestational age not mentioned	TXA PO 1950 mg with misoprostol 800 mcg SL vs. placebo with misoprostol 800 mcg SL Primary outcome: blood loss	Adjunct use of oral TXA with misoprostol to treat PPH had similar clinical and acceptability outcomes when compared to treatment with misoprostol alone. Proportion of women with active bleeding controlled with trial drugs alone and no additional interventions was similar in both groups: 77 (60.2%) placebo; 74 (56.9%) TXA, *p* = 0.59). Use of other interventions to control bleeding, including uterotonics, did not differ significantly between groups. Median blood loss at PPH diagnosis was 700 mL in both groups. Reports of side effects and acceptability were similar in the two groups.
Dutta, 2017 [35] (Conference abstract only)	Tertiary care hospital in Nadia, West Bengal, India	To evaluate a new surgical technique (Dutta’s) to prevent PPH due to major degree placenta previa during c-section	Non-randomized trial	94 pregnant women with major degree placenta previa undergoing c-section	Injection TXA 1 g IM + oxytocin 10 IU IV infusion Primary outcome: blood loss	Simple, safe, quick, effective procedure: intraoperative blood loss less than 300 cc in 89 (94.68%) cases. It reduces perfusion pressure, permits time for further steps. This technique is suitable for rural-based hospital in absence of adequate blood transfusion facility.
Joudeh, 2021 [36]	22 District Hospitals in Bihar, India	To assess PPH diagnoses and management, hypertensive disorders of pregnancy, birth asphyxia, and low birth weight, as part of the CARE’s AMANAT program (Comprehensive Emergency Obstetric and Neonatal Readiness.)	Non-randomized trial	11,259 pregnant women (diagnosis analysis) and 11,800 pregnant women (management analysis)	Physicians and nurse mentors conducted clinical instruction, simulations (PRONTO International curriculum & training kits) and teamwork and communication activities, infrastructure and management support, and data collection for 5 days weekly during 6 consecutive months. PPH management: IV fluids, uterotonics, TXA Primary outcome: level of PPH diagnosis and management	Lower level of PPH diagnosis than expected. But among PPH patients, 96% received fluids, 85% received uterotonics and 11% received TXA. There was a significant positive trend in the number of patients receiving TXA for PPH (6% to 13.8%, *p* trend = 0.03)
Khaing, 2021 [22] (Conference abstract only)	Central Women’s Hospital, Mandalay, Myanmar	To evaluate prophylactic TXA effectiveness	RCT Blinding not mentioned	220 pregnant women at low risk of PPH, vaginal delivery	TXA 1 g IV & oxytocin 10 IU IV vs. oxytocin 10 IU IV (without TXA) Primary outcome: blood loss	Mean total blood loss was significantly lower in the intervention group (213.1 ± 85.9 mL) than the control group (365.6 ± 203.4 mL). The mean measured blood loss from fetal delivery to 2 h postpartum was significantly lower in the intervention group (173.1 ± 56.0 mL) than the control group (227.7 ± 83.3 mL). The need of additional uterotonic drugs was significantly lower in the intervention group.
Li, 2018 [38]	Hospitals in Nigeria, Pakistan	To assess the cost-effectiveness of early administration (within 3 h after birth) of TXA added to usual care to treat PPH	Cost-effectiveness analysis using decision tree model & health-care provider perspective	No detail regarding Nigeria and Pakistan trial population (the trial recruited in total 20,000 women from 21 countries)	Primary outcome: costs (calculated in 2016 US$), life-years, and quality-adjusted life-years (QALYs) with and without TXA, incremental cost-effectiveness ratios (ICERs)	Intervention highly cost-effective in Nigeria and Pakistan: 0.18 QALYs at an additional cost of $37.12 per patient in Nigeria and an average gain of 0.08 QALYs at an additional cost of $6.55 per patient in Pakistan. The base case ICER results were $208 per QALY in Nigeria and $83 per QALY in Pakistan. These ICERs were below the lower bound of the cost-effectiveness threshold range in both countries.
McClure, 2015 [41]	Sub-Saharan African countries, unspecified Homes, clinics, and hospitals level of care	To determine the impact of TXA on PPH-related maternal mortality in sub-Saharan Africa	Mathematical model populated with baseline birth rates and mortality estimates based on a review of current interventions for PPH in sub-Saharan Africa, assuming 30% efficacy of TXA to reduce PPH; the model assessed prophylactic and treatment TXA use for deliveries at homes, clinics, and hospitals.	Not applicable	Not applicable Primary outcome: reduced maternal mortality ratio	With TXA only in the hospitals, less than 2% of the PPH mortality would be reduced. However, if TXA were available in the home and clinic settings for PPH prophylaxis and treatment, a nearly 30% reduction (nearly 22,000 deaths per year) in PPH mortality is possible. Given its feasibility to be given in the home, TXA can save many lives.
Mirghafourvand, 2013 [23]	Alzahra hospital, Tabriz, Iran	To determine the effect of prophylactic TXA on calculated and measured blood loss	Double-blind RCT	120 women with a term (38–42 weeks) singleton pregnancy at PPH low-risk, vaginal delivery	TXA 1 g IV & oxytocin 10 IU IV vs. placebo IV & oxytocin 10 IU IV Primary outcome: blood loss	Prophylactic TXA reduces blood loss after vaginal delivery in women with a low PPH risk. The mean (SD) calculated total blood loss (519 (320) vs. 659 (402) mL, *p* = 0.036) and measured blood loss from placental delivery to 2 h postpartum (69 (39) vs. 108 (53) mL, *p* < 0.001) was significantly lower in the intervention group. The frequency of calculated blood loss > 1000 mL was lower in the TXA group (7% vs. 18%, *p* = 0.048)
Naeiji, 2021 [24]	Shahid Beheshti University of Medical Science (SBUMS), Tehran, Iran	To evaluate the efficacy and safety of preoperative administration of IV TXA on blood loss during and after elective c-section.	Double-blind RCT	200 pregnant women with elective c-section. Gestational age not mentioned	TXA 1 to 1.5 g IV vs. distilled water before incision Primary outcome: intra-operative and post-operative blood loss and hemoglobin	Prophylactic use of IV TXA decreases the blood loss safely in women undergoing elective c-section: TXA decreased the mean blood loss by 25.3%. Mean volume of intra-operative blood loss was 391.1 (±67.4) mL in TXA group and 523.8 (±153.4) mL in control group which was statistically significant lesser with a 132.7 mL difference. Rate of >1000 mL and >500 mL bleeding and need to blood transfusion were also statistically significant lower in TXA group. Mean hemoglobin level was statistically significant lower in placebo group (11.77 ± 0.50 versus 11.31 ± 0.56) 6 h after c-section. No adverse reaction was documented.
Nargis, 2020 [25]	IBN SINA Medical College Hospital, Dhaka, Bangladesh	To evaluate the effectiveness IV TXA on blood loss in elective c-section	Double-blind RCT	120 pregnant women pregnant women with elective c-section after 35 weeks	TXA 1 IV vs. distilled water immediately after delivery of baby Primary outcome: intra-operative and post-operative blood loss and hemoglobin	Prophylactic use of IV TXA decreased blood losses from both placental deliveries to the end of c-section and from end of c-section to 2 h postpartum were significantly lower in the study group (*p* < 0.05). Total amount of oxytocin required was significantly less in TXA group (*p* < 0.05) also the number of women requiring other uterotonics (injectable methyl ergometrine, injectable carboprost and misoprostol per rectum) was significantly less in TXA group (*p* < 0.05). The amount of intra-operative fluid required were significantly less in TXA group (*p* < 0.005).
Nwabueze, 2021 [26] (Conference abstract only)	Federal Teaching Hospital Abakaliki (FETHA), Nigeria	To evaluate the efficacy of TXA at reducing blood loss following vaginal delivery	Double-blind RCT	Women undergoing vaginal births; sample size not mention.	TXA vs. placebo. Posology not mentioned Primary outcome: blood loss	IV TXA following vaginal delivery reduced blood loss. It reduced the need for additional uterotonics to control blood loss. However, blood loss greater than 500 was not significantly reduced: the mean estimated blood loss was significantly lower in the TXA group compared with the placebo group (174.87 ± 119.84 mL versus 341.07 ± 67.97 mL respectively; *p* < 0.0001). Additional uterotonics was required more in the control group compared to the treatment group 14 (16.67%) versus 3 (3.85%) of the treatment group, *p*-value of 0.007. There were no major complications noticed in the treatment group.
Oseni, 2021 [27]	Aminu Kano Teaching Hospital, Kano, Nigeria	To evaluate the effectiveness IV TXA on blood loss.	Double-blind RCT	244 pregnant women 37–42 weeks with emergency c-section	Pre-incision: TXA 1 g IV vs. normal saline water. Oxytocin in both groups Primary outcome: intra-operative and post-operative blood loss and hemoglobin	Significant reduction in blood loss TXA group: the average intraoperative blood loss was 414.0 mL in the study group and 773.8 mL in the control group (t = −16.18, *p* ≤ 0.01).
Resch, 2020 [39] (Conference abstract only)	Different levels of care Uttar Pradesh, India	To develop a PPH cost-effectiveness model to estimate the potential health impact and cost-effectiveness of a quality improvement program for PPH management featuring a first response bundle and a set of refractory PPH interventions in health facilities	Decision tree model to compare the status quo delivery of PPH care in two scenarios	1 million women delivering at home, subcenters, primary-health clinics, community-health centers, and district hospitals	Status quo PPH care: IV fluids, uterotonics, and uterine massage delivered with a setting-specific probability that increases with the level of health facility Strengthened PPH care: status quo interventions with TXA in all PPH cases, plus manual placenta removal and suturing when indicated. Enhanced scenario: further enhanced through implementation of non-surgical interventions for managing refractory PPH (including uterine balloon tamponade, aortic compression, and non-pneumatic anti-shock garment).	Implementation of an enhanced PPH care program, including the first response bundle and non-surgical refractory PPH interventions, is likely to be cost-effective and lifesaving in Uttar Pradesh, India (reduced PPH-related maternal mortality in intervention facilities by 98%, from 10.7 to 0.3 per 100,000 deliveries, averting 450 deaths per year). Moreover, enhanced PPH care is likely to generate more health impact and cost-savings compared with strengthened PPH care because of the greater reduction in number of surgeries needed.
Sahu, 2019 [37]	Referral hospital situated at the tribal terrain of Chhattisgarh, India	To evaluate the effectiveness IV TXA on blood loss.	Non-randomized trial	100 singleton pregnant women 35–42 weeks with elective and emergency c-section	Pre-incision: TXA 1 g IV vs. no TXA. Both groups received oxytocin 10 IU post baby delivery. Primary outcome: blood loss	Significant reduction in blood loss in TXA group: the mean blood loss (intra as well as postoperative) was 436.5 ± 118.07 mL in the study group in comparison to 616.5 ± 153.34 mL in the control group (*p* ≤ 0.05)
Sujata, 2016 [28]	Max Hospital, New Delhi, India	To evaluate the effectiveness IV TXA on blood loss in c-section among women at high PPH risk	Single-blinded RCT	60 singleton pregnant women with elective or emergency c-section: gestational age not mentioned.	Per-op: TXA 1 g IV vs. normal saline water. Primary outcome: need for additional uterotonics	Significant reduction in blood loss in TXA group: uterotonic drugs were required in 7 (23%) patients assigned to TXA and 25 (83%) patients in the control group (*p* < 0.001)
Tabatabaie, 2021 [29]	Dr. Ali Shariati and Persian Gulf Hospitals of Bandar Abbas, Iran	To compare the effect of TXA and misoprostol on blood loss during and after c-section	Triple-arm RCT, non-blinded	300 singleton pregnant women, 37–42 weeks	Group A: TXA 10 mg/kg IV; Group B: misoprostol 600 mcg rectal; Group C: 200 mL normal saline. All groups received oxytocin. Primary outcome: blood loss	Both medicines are effective in reducing the amount of blood loss during c-section with misoprostol being more effective than TXA. Level of blood loss in ml: 444.70 ± 100.58 (TXA); 299.98 ± 162.79 (misoprostol); 568.84 ± 147.07 (placebo), *p* < 0.001
Tali, 2016 [30] (Conference abstract only)	Jose R. Reyes Memorial Medical Center, Manila, Philippines	To compare the effect of TXA on blood loss in vaginal delivery	Double-blind RCT	Not mentioned	TXA 1 g IV & oxytocin 10 IU IV vs. placebo IV & oxytocin 10 IU IV Primary outcome: blood loss	The prophylactic use of TXA may reduce blood loss: the mean (SD) calculated total blood loss (167 (162) versus 463 (348) mL, *p* < 0.001), measured blood loss from fetus delivery to placental delivery (133 (47) versus 207 (66) mL, *p* < 0.001), from placental delivery to 2 h postpartum (82 (33) versus 136 (88) ml, *p* < 0.001). The frequency of calculated blood loss >1000 mL was lower in the TXA group (0% versus 12%, *p* < 0.001)
Zargar, 2018 [31]	Ahvaz Jundishapur University of Medical Sciences, Ahvaz, Iran	To compare the effect of TXA and prostaglandin analog on reducing PPH in cesarean or vaginal delivery.	Triple-blind RCT	248 singleton pregnant women, 38–40 weeks	TXA IV: 4 g for an hour and then 1 g over 6 h infusion vs. prostaglandin analog IM 0.25 mg up to 8 doses (Hemebate). Primary outcome: blood loss	TXA had comparable effects with prostaglandin analog on reducing PPH in women with uterine atony and in those undergoing C section or vaginal delivery: postoperative bleeding did not significantly differ between the two groups (68.2 ± 6.1 mL and 69.1 ± 175.73 mL, respectively, *p* = 0.6). Moreover, hemoglobin declines were 1 ± 0.4 g/dL and 1.2 ± 0.5 g/dL in TXA and prostaglandin group respectively, indicating that the difference was not statistically significant (*p* = 0.7)

IM: intramuscular; IU: international unit; IV: intravenous; PPH: postpartum hemorrhage; RCT: randomized controlled trial; SL: sublingual; TXA: tranexamic acid.

### 3.3. Patient Population

Most of the clinical trials enrolled women with a singleton pregnancy who delivered vaginally or by cesarean section. Their sample sizes were relatively small to moderate (n = 40 to 300) [29,32] except in the studies by Abdel-Aleem (n = 740) [18] and Joudeh (n > 12,000) [36].

The cost-effectiveness study by Resch included 1 million women [39]. Li did not provide such information [38]. McClure’s mathematical model used baseline birth rates and mortality estimates drawing from on a review of existing PPH interventions in sub-Saharan Africa [40].

### 3.4. Context

Most clinical and non-clinical studies took place in tertiary care and often university hospitals in the country capital or a major urban setting. Exceptions include studies by Joudeh (district hospitals) [36], McClure (homes and clinics in addition to hospitals) [41], and Resch (homes, community health centers, primary health clinics, and district hospitals) [39].

Out of the 23 studies, only McClure’s included low-income countries (its mortality modeling focused on all sub-Saharan Africa—without specific mention of countries, which presumably encompassed low-income and middle-income countries) [41]. The other studies focused on lower-middle-income countries (see Table 1).

### 3.5. Concepts

As quantitatively described in Table 2 and further expanded in Table 4, the concept most frequently studied was the effectiveness of TXA not only to treat PPH (9 references) but as well prevent it (16 references). McClure’s mathematical model also examined TXA effectiveness but in decreasing PPH-related maternal mortality. Financial feasibility [20,38,39] and operational feasibility in terms of commodity security [20], health staff capacitation [36], and service delivery [39] were addressed by a handful of studies. The acceptability of TXA by women was only examined by Diop [21]. None of the studies looked at the perspectives of providers in terms of acceptability, nor did they examine the health system components related to governance and health information systems.

#### 3.5.1. Effectiveness

##### PPH Treatment

As a medication to treat PPH, TXA was found effective for both vaginal and cesarean births in the studies by Ajroudi [32], Briki (in combination with fibrinogen) [33], and Dimassi (in combination with sulprostone) [34]. Diop found that the addition of sublingual TXA to misoprostol resulted in similar clinical and acceptability outcomes in vaginal births compared to treatment with misoprostol alone [21]. In a large study involving 22 district hospitals in Bihar, India, Joudeh assessed the effectiveness of a comprehensive emergency obstetric and neonatal readiness program, which included TXA in its PPH management package [36]. The study results showed an insufficient level of PPH diagnosis but a significantly positive trend in the number of patients diagnosed with PPH who received TXA (from 6% to 14%).

##### PPH Prevention

For use in preventing PPH, TXA was reported to be effective for both vaginal and cesarean births. As an example, in their study with a large sample size of women planned for elective cesarean birth, Abdel-Aleem found that the administration of TXA 1 g intravenously 10 min before the procedure resulted in significantly reduced blood loss (241 mL vs. 510 mL in the control group) [18]. There were, however, no differences in other outcomes of interest, such as additional uterotonic use, additional surgical interventions, admission to the intensive care unit, or hospitalization length. The study was not powered to assess TXA efficacy in preventing severe PPH (≥1000 mL) or safety, especially thromboembolic complications.

Compared to sublingual misoprostol, TXA was shown by Bose to prevent more post-cesarean bleeding in women without high risk factors for PPH, but not in women with such risk factors [20]. Tabatabaie found that TXA-oxytocin and misoprostol-oxytocin combinations were both effective in preventing post-cesarean blood loss with misoprostol-oxytocin being more effective [29]. Finally, Zargar showed that TXA had comparable effects as a prostaglandin analog (Hemebate) in preventing post-cesarean bleeding [31].

##### Maternal Mortality

McClure’s model in sub-Saharan countries estimated the following proportions of births occurring in different locations: 15% in hospitals, 35% in health clinics, and 50% at home [41]. The study showed that if TXA is available for PPH prophylaxis and treatment only in hospital settings, less than 2% of PPH-related mortality would be reduced. In contrast, if it is availed in home and health clinic settings, where the majority of births occur, a reduction in PPH mortality of nearly 30% (almost 22,000 deaths per year) would be possible. (Note that in the WOMAN trial and subsequent systematic review, Shakur concluded that intravenous TXA given immediately after bleeding onset reduced primary PPH mortality by 19% [8,9]).

#### 3.5.2. Feasibility

Li showed that the use of TXA for PPH treatment could yield an average gain in quality-adjusted life-years (QALYs) of 0.18 at an additional cost of $37.12 per patient in Nigeria and 0.08 at an additional cost of $6.55 per patient in Pakistan [38]. The best-case results for incremental cost-effectiveness ratios (ICERs) were $208 per QALY in Nigeria and $83 per QALY in Pakistan, considered highly cost-effective in both countries.

In Kerala, India, Bose found TXA to be more effective than misoprostol for PPH prevention in cesarean births [20]. They reported the unit cost for each medication: INR (Indian Rupee) 52 for misoprostol—made by Cipla and INR 57 for TXA—made by Ozone. However, they did not perform a comparative economic analysis. The authors still thought that misoprostol might be operationally more feasible in resource-restricted settings in India as it does not require refrigeration, in addition to being freely available from government supplies.

In Uttar Pradesh, India, Resch showed that the use of TXA in all PPH cases would be the most cost-effective if it is integrated into a PPH care package enhanced by non-surgical interventions [39]. These interventions would help manage refractory PPH and include uterine balloon tamponade, aortic compression, and non-pneumatic anti-shock garment. The components of such an enhanced bundle would be implemented in adequation with the health facility level. Taken together, the components of this bundle could generate greater health impact and cost-savings thanks to a larger reduction in the number of required surgeries. In Uttar Pradesh, this would yield an estimated 98% reduction of PPH-related mortality (from 10.7 to 0.3 per 100,000 deliveries), averting 450 deaths yearly.

In the hospital settings in Bihar, India, the increased number of PPH patients who received TXA in the comprehensive emergency obstetric and neonatal readiness program would not have been possible without substantial investment in staff capacitation, as described by Joudeh [36]. It was the result of a high-intensity training and coaching program involving mentors (physicians and nurses), who offered training and supportive supervision to clinicians five days weekly over six consecutive months.

#### 3.5.3. Acceptability

Diop found that the adjunct use of oral TXA with misoprostol to treat PPH had similar acceptability and clinical outcomes when compared to treatment with misoprostol alone [21]. The acceptability reported by women referred to the side effects they experienced, such as shivering, fever, nausea, vomiting, diarrhea, and fainting in decreasing frequency.

## 4. Discussion

Our rapid scoping review comprised 20 TXA effectiveness studies, 2 economic evaluations, and 1 mortality modeling. All the studies were undertaken in lower-middle-income countries except for the mortality study, which included unspecified low-income countries in sub-Saharan Africa. Most of the studies occurred in tertiary CEmOC referral settings. Overall, TXA was found to be effective in both treating and preventing PPH in vaginal and cesarean birth when compared to placebo or other medications. It has the potential to decrease PPH-related deaths if made available in home and clinic settings. It is potentially cost-effective, notably when integrated into a PPH intervention package enhanced with non-surgical interventions to manage refractory PPH. Capacitating service providers to increase the use of TXA to treat PPH appears to require time-intensive training and supportive supervision but would be necessary for sustainable integration into health service delivery. One study found the acceptability of TXA side effects comparable to that of misoprostol. There were no studies examining TXA acceptability from providers’ perspectives and its implications for health governance and health information systems.

The outcomes of the identified PPH treatment studies align with those of the WOMAN trial, which also included low-income countries [8]. It is well established that countries belonging to the low-income group and those beset by fragility and humanitarian disasters carry the largest burden of global maternal fatalities [2]. McClure showed a potential maternal mortality decrease in rolling out TXA in sub-Saharan Africa, especially when it is made available for deliveries in lower levels of facility-based and home-based maternity care [41]. The model may overestimate the reduction in PPH mortality, as it is based on 50% of births occurring at home in sub-Saharan Africa. This figure contrasts with the 31% estimated in the same region using data from 2000 to 2019 [42]. Disaggregated data from the latter study indicate that the countries with the highest proportion of home births in sub-Saharan Africa were Chad (78%), Ethiopia (73%), Niger (70%), Madagascar (64%), Nigeria (59%), and Angola (53%) (there was no data for South Sudan, but another study done at a county level found that 72% of births occurred at home [43]). In countries with such a high proportion of home births, the availability and safe administration of TXA may play a critical role in reducing a third or more of PPH mortality according to McClure’s model. However, based on current clinical recommendations, this would require the presence of staff competent in injecting TXA. Therefore, oral TXA could be a game-changer if found safe and effective in treating PPH. Notwithstanding, our review identified limited information on the safety, feasibility, acceptability, and effectiveness of TXA in these lower and often resource-challenged levels of care. Piloting and evaluating the implementation of TXA by trained health care professionals in such contexts would be needed. However, this should be done as part of an overall health system strengthening strategy focused on curbing maternal death and disability. As shown by a secondary analysis of the WOMAN trial, which examined the deaths of 483 women following PPH, other clinical and contextual factors should be tackled, including maternal anemia, delayed referral to higher levels of care, unavailability of blood transfusion, and poor facility infrastructure [44].

With regard to maternal anemia, it is prevalent in low-resource settings and has been long established to affect negatively women experiencing postpartum blood loss, even in a small amount [45]. Maternal anemia is associated with increased risk of PPH, low birthweight, small-for-gestational age babies, and perinatal death [46]. Therefore, preventing PPH is critical for women living in these settings and has been prioritized in numerous studies, including those identified in our scoping review. Owing to the mixed quality of the extant literature, WHO is not recommending TXA for PPH prevention and is awaiting the results the WOMAN-2 trial [47]. The WOMAN-2 trial is a large randomized double-blind controlled trial taking place in Pakistan and Zambia.

In terms of health system considerations useful for TXA planning and implementation in low-resource settings, the scoping review may offer some insights to decision-makers and practitioners on cost-effectiveness, health staff training and support, and integration of TXA into PPH care packages. It would be helpful to document how TXA can be effectively integrated into (i) national technical guidance and health policies, (ii) clinical records and the health information system, and (iii) lower-level maternity care settings with limited infrastructure and staff capacity. 

Rapid scoping reviews, by their very nature, have flaws since they focus over a shorter period on finding knowledge gaps and repercussions for decision-making, as well as guiding future research. [17]. Therefore, biases may have been introduced in literature searches, article retrieval, and assessment. We may have missed key papers by restricting our search to English, French, and Spanish articles and speeding the data extraction procedure. We did not assess the methodological quality of the studies and cannot comment on their scientific robustness. The lists of references in the publications may not have been fully scanned due to time constraints, and we did not contact authors for further information. 

## 5. Conclusions

TXA could be a cost-effective addition to the PPH treatment toolkit in vaginal and cesarean births in hospital settings of lower-middle-income countries. Information on how to prepare the health system and health services to integrate TXA in lower-level maternity care facilities in low-resource settings is scarce. Further implementation research is needed to assist decision-makers and practitioners in developing a TXA-inclusive PPH treatment package to limit bleeding-related deaths and disabilities among women giving birth in resource-challenged settings.

## Figures and Tables

**Table 1:** Eligibility criteria.

Study Design & Publication Type	Randomized Controlled Trials; Non-Randomized Trials; Peer-Reviewed (No Grey Literature)
Timeline	Published between 1 January 2011 and 15 September 2021
P (population)	Women who had a vaginal or cesarean birth
C (concept)	Postpartum hemorrhage; feasibility; acceptability; health system considerations
C (context)	Low-income countries; lower-middle-income countries

**Table 2:** Report types.

Item (n = 23)		Count	%		Comment
Publication type					
	Full-text articles	15	65		Abdel-Aleem, Bose, Briki, Diop, Joudeh, Li, McClure, Mirghafourvand, Naeiji, Nargis, Oseni, Sahu, Sujata, Tabatabaie, Zargar
	Conference abstracts	8	35		Agrawal, Ajroudi, Dimassi, Dutta, Khaing, Nwabueze, Resch, Tali
Study country					
	Low-income	1	4		Unspecified countries in sub-Saharan Africa (McClure)
	Lower-middle-income	23	100		
	India	6	26		Bose, Dutta, Joudeh, Resch, Sahu, Jujata,
	Iran	4	17		Mirghafourvand, Naeiji, Tabatabaie, Zargar
	Tunisia	3	13		Ajroudi, Briki, Dimassi
	Nigeria	3	13		Li, Nwabueze, Oseni
	Others	9	39		Bangladesh (Nargis), Egypt (Abdel-Aleem) Myanmar (Khaing), Nepal (Agrawal), Pakistan (Li), Philippines (Tali), Senegal (Diop), Vietnam (Diop), unspecified countries in sub-Saharan Africa (McClure)
Study type					
	Effectiveness for PPH	20	87		
	Prevention in cesarean birth	11	55		Abdel-Aleem, Agrawal, Bose, Dutta, Naeiji, Nargis, Oseni, Sahu, Sujata, Tabatabaie, Zargar
	Prevention in vaginal birth	5	25		Khaing, Mirghafourvand, Nwabueze, Tali, Zargar
	Treatment in cesarean birth	4	20		Ajroudi, Briki, Dimassi, Joudeh
	Treatment in vaginal birth	5	25		Ajroudi, Briki, Dimassi, Diop, Joudeh
	Economic evaluation	2	9		Li, Resh
	Maternal mortality modeling	1	4		McClure
Outcome of interest					
	Acceptability	1	4		Diop
	Feasibility (operational)	3	13		Bose, Joudeh, Resch
	Feasibility (financial)	3	13		Bose, Li, Resh

**Table 4:** Contexts and main concepts of included studies.

Study & Year	Countries		Levels of Care		Outcomes of Interest			Health System Environment					
	Low-Income	Lower Middle-Income	BEmOC	Hospital CEmOC	Feasibility	Acceptability	Effectiveness	Governance & Policy Alignment	Procurement & Commodity Security	Health Staff Awareness, Motivation & Training	Service Delivery	Health Information System	Financing
Abdel-Aleem, 2013 [18]	-	✓	-	✓	-	-	✓	-	-	-	-	-	-
Agrawal, 2018 [19]	-	✓	-	✓	-	-	✓	-	-	-	-	-	-
Ajroudi, 2015 [32]	-	✓	-	✓	-	-	✓	-	-	-	-	-	-
Bose, 2017 [20]	-	✓	-	✓	✓	-	✓	-	✓	-	-	-	✓
Briki, 2018 [33]	-	✓	-	✓	-	-	✓	-	-	-	-	-	-
Dimassi, 2018 [34]	-	✓	-	✓	-	-	✓	-	-	-	-	-	-
Diop, 2020 [21]	-	✓	-	✓	-	✓ ^a^	✓	-	-	-	-	-	-
Dutta, 2017 [35]	-	✓	-	✓	-	-	✓	-	-	-	-	-	-
Joudeh, 2021 [36]	-	✓	-	✓	✓	-	-	-	-	✓	-	-	-
Khaing, 2021 [22]	-	✓	-	✓	-	-	✓	-	-	-	-	-	-
Li, 2018 [38]	-	✓	-	✓	✓	-	-	-	-	-	-	-	✓
McClure, 2015 [41]	✓	✓	✓ ^b^	✓	-	-	✓ ^c^	✓	-	-	-	-	-
Mirghafourvand, 2013 [23]	-	✓	-	✓	-	-	✓	-	-	-	-	-	-
Naeiji, 2021 [24]	-	✓	-	✓	-	-	✓	-	-	-	-	-	-
Nargis, 2020 [25]	-	✓	-	✓	-	-	✓	-	-	-	-	-	-
Nwabueze, 2021 [26]	-	✓	-	✓	-	-	✓	-	-	-	-	-	-
Oseni, 2021 [27]	-	✓	-	✓	-	-	✓	-	-	-	-	-	-
Resch, 2020 [39]	-	✓	✓	✓	✓	-	✓	-	-	-	✓	-	✓
Sahu, 2019 [37]	-	✓	-	✓	-	-	✓	-	✓	-	-	-	-
Sujata, 2016 [28]	-	✓	-	✓	-	-	✓	-	-	-	-	-	-
Tabatabaie, 2021 [29]	-	✓	-	✓	-	-	✓	-	-	-	-	-	-
Tali, 2016 [30]	-	✓	-	✓	-	-	✓	-	-	-	-	-	-
Zargar, 2018 [31]	-	✓	-	✓	-	-	✓	-	-	-	-	-	-

✓: concept found in article; -: concept not found in article; ^a^: acceptability in terms of side-effects; ^b^: including home birth; ^c^:effectiveness in terms of reducing MMR; BEmOC: basic emergency obstetric care; CEmOC: comprehensive emergency obstetric care.

## Data Availability

Not applicable.

## Appendix A

Appendix A contains the PRISMA statement, the protocol, low-income and lower-middle-income groups as defined by the World Bank, and the MEDLINE search strategy.
